# Dystonia Diagnosis: Clinical Neurophysiology and Genetics

**DOI:** 10.3390/jcm11144184

**Published:** 2022-07-19

**Authors:** Lazzaro di Biase, Alessandro Di Santo, Maria Letizia Caminiti, Pasquale Maria Pecoraro, Simona Paola Carbone, Vincenzo Di Lazzaro

**Affiliations:** 1 Neurology Unit, Campus Bio-Medico University Hospital Foundation, Via Álvaro del Portillo 200, 00128 Rome, Italy; a.disanto@unicampus.it (A.D.S.); m.caminiti@unicampus.it (M.L.C.); p.pecoraro@unicampus.it (P.M.P.); simonapaola.carbone@unicampus.it (S.P.C.); v.dilazzaro@policlinicocampus.it (V.D.L.); 2 Unit of Neurology, Neurophysiology, Neurobiology, Department of Medicine, Campus Bio-Medico University of Rome, Via Álvaro del Portillo 21, 00128 Rome, Italy; 3 Brain Innovations Lab., Campus Bio-Medico University of Rome, Via Álvaro del Portillo 21, 00128 Rome, Italy

**Keywords:** dystonia, clinical diagnosis, neurophysiology, genetics

## Abstract

Dystonia diagnosis is based on clinical examination performed by a neurologist with expertise in movement disorders. Clues that indicate the diagnosis of a movement disorder such as dystonia are dystonic movements, dystonic postures, and three additional physical signs (mirror dystonia, overflow dystonia, and geste antagonists/sensory tricks). Despite advances in research, there is no diagnostic test with a high level of accuracy for the dystonia diagnosis. Clinical neurophysiology and genetics might support the clinician in the diagnostic process. Neurophysiology played a role in untangling dystonia pathophysiology, demonstrating characteristic reduction in inhibition of central motor circuits and alterations in the somatosensory system. The neurophysiologic measure with the greatest evidence in identifying patients affected by dystonia is the somatosensory temporal discrimination threshold (STDT). Other parameters need further confirmations and more solid evidence to be considered as support for the dystonia diagnosis. Genetic testing should be guided by characteristics such as age at onset, body distribution, associated features, and coexistence of other movement disorders (parkinsonism, myoclonus, and other hyperkinesia). The aim of the present review is to summarize the state of the art regarding dystonia diagnosis focusing on the role of neurophysiology and genetic testing.

## 1. Introduction

Dystonia is a term used to identify hyperkinetic movement disorders in which dystonia is the prominent feature. However, dystonia can also be present in other conditions. According to the etiology, dystonia can be distinguished as acquired, inherited, or idiopathic. The diagnosis of dystonia is based on clinical examination conducted by physicians with expertise in movement disorders through a careful examination of the phenomenology of the condition that allows for a classification of dystonia. For the diagnosis of dystonia syndrome, the examiner should follow the definition of dystonia approved in the last expert consensus [1], articulated in three subdefinitions:

1. Dystonia is a movement disorder characterized by sustained or intermittent muscle contractions causing abnormal, often repetitive, movements, postures, or both.
2. Dystonic movements are typically patterned, twisting, and may be tremulous.
3. Dystonia is often initiated or worsened by voluntary action and associated with overflow muscle activation.

The examiner should focus on the classic five physical signs of dystonia syndromes: two main physical signs (dystonic movements and dystonic posture) and three additional physical signs (mirror dystonia, overflow dystonia and geste antagonists/sensory tricks) [2,3].

The role of laboratory analysis, neuroimaging studies, neurophysiology, and genetic tests is to support the etiology definition of the disease, according to the Axis II of Dystonia classification [1,4]. 

The aim of the present review is to summarize the state of the art regarding dystonia diagnosis focusing on the role of neurophysiology and genetic testing.

## 2. Clinical Neurophysiology

Clinical neurophysiology techniques such as EMG mapping [2,5] allow clinicians to support the diagnosis of dystonia and to explore the activity of individual muscles which is not always easy to achieve with a clinical inspection alone. In addition, clinical neurophysiology with different techniques, such as transcranial magnetic stimulation (TMS) [6,7], transcranial direct current stimulation (tDCS) [8,9], or the newest transcranial focused ultrasound stimulation (tFUS) [10,11,12], allow clinicians to explore in a non-invasive way the brain functions In recent years, these techniques have been widely used as tools to characterize distinctive features and improve diagnostic accuracy for different movement disorders [13], particularly parkinsonian syndromes [14,15,16], tremor syndromes [17,18,19], myoclonus [20], and dystonia [21]. The literature includes several studies that use different neurophysiological tests to assess dystonia [22] (Table 1). Despite the amount of evidence, most of the studies on dystonia neurophysiology have a small sample size and focus on specific forms of dystonia (e.g., DYT-TOR1A); therefore, results are not always generalizable to all forms of dystonia. Neurophysiology assessment is not formally included in the diagnostic process [1]; however, neurophysiological tests can support the diagnosis.

Since the early 1980s, neurophysiology has been used to characterize dystonia pathophysiology. Most studies were performed in focal hand dystonia (FHD) [22]. At first, dystonia was classified as a basal ganglia (BG) disorder; however, in recent years, evidence points to a disorder arising from a complex network system involving the cerebral cortex (motor and sensory area), the basal ganglia, the brainstem, and the cerebellum [43,44], suggesting that is it possible that several structures could be simultaneously involved in the pathogenesis of dystonia subtypes [43,44].

The electromyographic (EMG) pattern observed in dystonia patients records simultaneous activation of agonist and antagonist muscles (co-contraction), prolonged duration of EMG bursts, and involuntary overflow activation of muscles not directly involved in the movement [3,23].

The most relevant neurophysiological feature shared by all dystonia subtypes is the reduced inhibition of central motor circuits [22]. This is demonstrated by characteristics in several structures: (1) at the subcortical level, a reduction of presynaptic inhibition in the spinal cord has been reported in patients with FHD [24]; (2) at the brainstem level, - a reduced inhibition in the blink reflex recovery cycle in blepharospasm patients [25] and an impairment of the trigeminocervical reflex produced by infraorbital nerve stimulation in torticollis patients was noted [26]; and (3) at the motor cortex level, a loss of inhibition was demonstrated with several transcranial magnetic stimulation (TMS) protocols. Several studies reported abnormalities in dystonic patients of paired pulse protocol as short intracortical inhibitions (SICI), that is, an inhibition of motor cortex response produced by a subthreshold conditioning stimulus followed by a supra-threshold stimulus. SICI is reduced in different subtypes of dystonia [27,28,29]. Reduced transcallosal inhibition was also demonstrated in FHD patients with mirror dystonia. In these patients, stimulation of one hemisphere does not suppress motor responses evoked by a stimulus delivered about 10 ms later over the contralateral hemisphere, as observed in normal subjects [30]. Finally, the duration of the cortical silent period (SP), the inhibition of ongoing muscular activity produced by a TMS pulse during muscle contraction, is reduced in dystonic patients [31], and the lack of suppression could be related to some specific tasks [32].

In recent years, the relevance of the cerebellum in dystonia’s pathophysiology has been investigated [45]. The eye blink classic conditioning (EBCC) protocols consist of electric stimulation of the supraorbital nerve. This protocol that involves cerebellar circuits shows impairment in focal dystonia patients [33], while it is normal in inherited dystonia caused by the DYT-TOR1A and DYT-THAP1 gene mutation [34]. A further test evaluates the motor cortex inhibition produced by cerebellar stimulation. In control subjects, stimulation of one cerebellar hemisphere produces a suppression of the contralateral motor cortex at intervals between 5 and 10 ms [46]. Cerebellar inhibition is impaired in dystonic patients [35].

Traditionally, dystonia was referred to as a motor disorder; however, several recent studies have provided evidence on the role of the somatosensory system in dystonia pathogenesis. Several studies suggested that abnormalities in the somatosensory system are present in almost all dystonic patients, and several neurophysiology tests investigated these findings. The most relevant discovery is the abnormality in the somatosensory temporal discrimination threshold (STDT) [37]. STDT represents the shorter interval at which two different stimuli are perceived as separate. Cervical dystonia (CD) patients have abnormally increased STDT, and the effect seems higher in CD patients with tremor. In a validation study, 51 CD were compared to essential tremor (ET) patients and Parkinson’s disease (PD) patients. The authors found that compared to ET patients, if STDT is ≤67 ms, it has 100% sensitivity and 100% negative predictive value, while if STDT is ≥120 ms, it has 100% specificity and 100% positive predictive value to differentiate ET from CD. However, no statistically significant differences were found between the PD and CD groups even though evidence suggests that STDT is normal in the early PD phase and becomes abnormal in later stages, while STDT is abnormally increased from the first stages of dystonia disease. Another important feature in dystonic patients is the somatosensory discrimination threshold tested with a grating orientation task (GOT) that is a measure of spatial tactile discrimination. These parameters results increased in all idiopathic forms of dystonia, while they are normal in inherited disease cases [36]. Proprioception is also altered in dystonic patients as demonstrated by an abnormally increased tonic vibration reflex (TVR) [38]. Moreover, a study demonstrated that dystonic patients have kinanesthesia impairment seen as abnormal perception of the Aristotle’s illusion, suggesting cortical impairment of somatosensory processes [47]. One possible cause of all these abnormalities could be a deficit in the lateral (or surround) inhibition process, as demonstrated by a somatosensory-evoked potential (SEPs) study [48].

Finally, another possible contribution to dystonic pathophysiology is represented by maladaptive plasticity. Abnormal sensory-motor plasticity was demonstrated using a paradigm termed paired associative stimulation (PAS) In this TMS protocol, cortical stimulation is paired with peripheral nerve stimulation at an interstimulus interval of 25 ms resulting in long-term potentiation-like phenomenon (LTP). This form of LTP is pathologically enhanced in FHD [39]. Maladaptive plasticity could be a key factor in the development of dystonic symptoms and a peculiar feature of dystonic patients as suggested by other studies that did not find the same increased plasticity in DYT-TOR1A carrier subjects [49] and in psychogenic dystonia patients [50]. A pronounced increase of PAS-related plasticity was also reported in Costello syndrome, a genetic syndrome characterized by pronounced dystonia [51,52]. Furthermore, evidence of abnormal plasticity in dystonic patients was highlighted with the use of high-frequency repetitive somatosensory stimulation (HF-RSS) [40]. HF-RSS is a repetitive electric stimulation delivered though surface electrodes on the skin that enhances inhibitory sensorimotor processes. In HS, it usually increases inhibition, while in CD patients inhibition is reduced.

Although all this evidence suggests that dystonia is a complex network disorder involving the brainstem, the basal ganglia, the thalamus, the cortex, and the cerebellum [44], originally dystonia was referred to as basal ganglia disease. Several trials point out that electrical modulation of the basal ganglia network through continuous deep brain stimulation (DBS) in internal globus pallidus (GPi) could improve generalized dystonia symptoms [53]. DBS electrodes were also used to invasively record synchronized neuronal activities, pointing out that in line with other movement disorders, pathological basal ganglia oscillatory activities [54] can be found in dystonic patients [41,42]. This invasive recording of local field potentials (LFP) of basal ganglia revealed that GPi and external globus palidus (GPe) have a decreased discharge rate and irregular firing in dystonic patients [55,56]. In addition, LFP studies demonstrated that pallidus nuclei of dystonic patients show excessive synchronized activities in the 4–10 Hz frequency band [42].

The study of oscillatory activities in neurological disorders [54] revealed new pathological biomarkers in recent years. Several authors suggested that these abnormalities could be used as biomarkers to deliver electrical DBS only in response to pathological neuronal oscillation (adaptative DBS-aDBS). This technique was mainly evaluated in Parkinson’s disease patients [57,58,59] in which LFP monitoring could be supported by multiparametric [60] motor symptoms monitoring [61,62,63] with the assistance of artificial intelligence algorithms [64]. It has been suggested that this protocol could be translated to dystonic patients with specific biomarkers, such as GPi LFPs theta-alpha band activity [41,42,53], in combination with dystonic muscle activity monitoring through subcutaneous EMG or wearable accelerometer devices [53].

## 3. Dystonia Genetics

**Figure 1:**
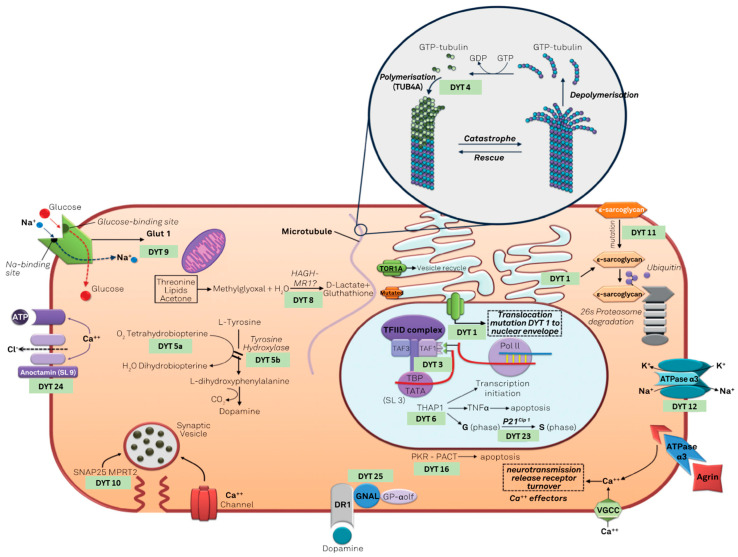
The “dystonia cell” describe the cellular pathway involved in genetic dystonias (modified under the terms and conditions of the Creative Commons Attribution (CC BY) license from [66]).

Dystonia genetics is a wide field with continuous updates. After the first description of DYT-TOR1A, several other genes have been proposed as linked with the dystonia phenotype [65]. As in other fields of genetics, after the first years focused on the genetic marker, the focus is moving on to proteomics, searching the causal link between the protein produced by these genes and the phenotype of dystonia. Camargos and Cardoso [66] proposed a model of the “dystonia cell” linking the dystonic genes to the proteins function (Figure 1), based mainly on the classic DYT nomenclature. 

**Figure 2:**
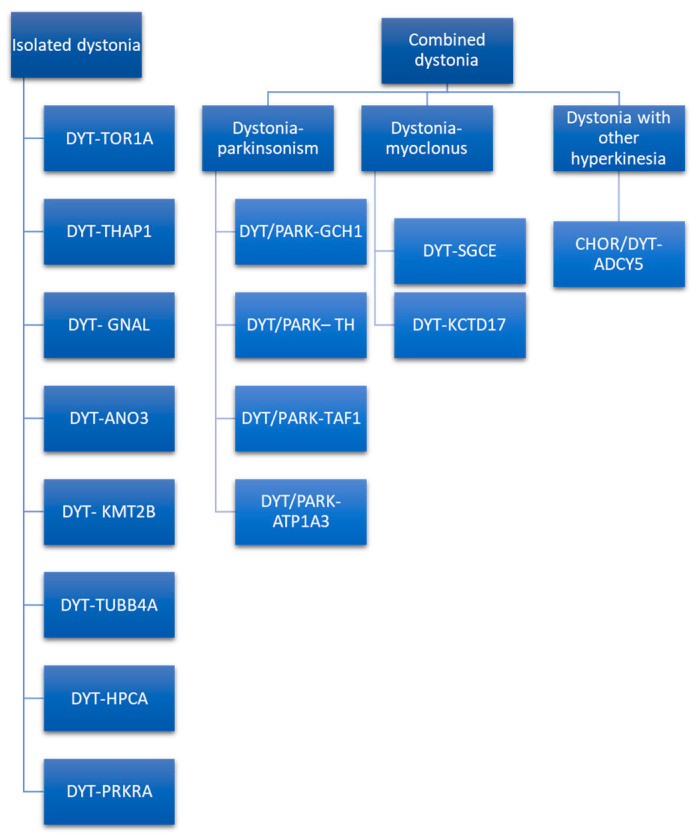
Isolated and combined genetic forms of dystonia [69].

The classic DYT nomenclature is based on locus symbols (e.g., DYT 1) and has been used for several years. It is still used in literature and clinical practice [67]. However, the system of locus symbols has been challenged by advances in techniques of genetics research that allow us to define the causative gene, as explained by Marras et al. [68], and the need to renovate the nomenclature system has arisen. The MDS Task Force for the Nomenclature of Genetic Movement Disorders proposed new recommendations, whose use in research and clinical practice is strongly encouraged [69]. This new nomenclature strictly connects the prefix to the predominant phenotype and considers the causative gene rather than the locus symbols (e.g., DYT 1 is now named DYT-TOR1A) [4]. The prefix DYT is used only if dystonia is the prominent disease feature due to a pathogenetic mutation [69]. Otherwise, if another movement disorder is a prominent feature along with dystonia, a double prefix would be assigned (e.g., DYT/PARK-ATP1A3). Indeed, genetic dystonia can be isolated or combined with other movement disorders such as parkinsonism, myoclonus, or other hyperkinesia (Figure 2).

Moreover, in the proposed nomenclature and in the last consensus update on dystonia, the term complex dystonia is used, referring to conditions in which dystonia predominates the clinical phenotype but occurs in the context of a complex disease including symptoms other than movement disorders [1,69]. For example, Wilson disease is named according to the proposed nomenclature with a DYT prefix (DYT-ATP7B), and the same happens for Lesch–Nyhan syndrome and other infantile and childhood onset disease [69]. Given that most of isolated hereditary dystonia is recognized as an autosomal dominant inheritance, the mode of transmission cannot be used as the only criterion to make a differential diagnosis. To guide the clinician towards a genetic diagnosis of dystonia, at least clinical phenotype and age of onset should be considered (Table 2). If dystonia dominates the clinical picture, one of the isolated dystonias may be considered, and the gene mutations involved may be DYT-TOR1A, DYT-THAP1, DYT-GNAL, DYT-ANO3, DYT-KMT2B, DYT-TUBB4A, DYT-HPCA, and DYT-PRKRA [70]. The last-mentioned dystonia is a controversial classification, as it is considered as combined dystonia by some authors [71] and as isolated dystonia by others [70]. Indeed, despite parkinsonism being described in about half the patients, it seemed to be caused not by true parkinsonian features, but by slow movements of dystonic body parts [70]. The isolated form of dystonia could be distinguished according to the age of onset, body distribution, temporal pattern, associated features, responses to drugs, response to DBS, and brain imaging. Regarding age of onset, in infancy, childhood, and adolescence DYT-TOR1A, DYT-THAP1, DYT-KMT2B, DYT-TUBB4A, DYT-PRKRA, and DYT-HPCA are more probable, while DYT-ANO3 and DYT-GNAL begin in early adulthood. In particular, DYT-ANO3 recognizes two peaks of the age of onset: one in infancy/childhood and one in early-late adulthood [70]. Age at onset may by modified by several aspects, e.g., penetrance as is the case of DYT-TOR1A [72]. Hence, age of onset alone cannot be used as the only criteria to orient the diagnosis. According to body distribution, generalized forms of isolated dystonia are mainly due to DYT-TOR1A, DYT-THAP1, DYT-KMT2B, DYT-HPCA, and DYT-PRKRA. Among these, DYT-TOR1A, DYT-HPCA, and DYT-KMT2B usually begin in the lower limbs asymmetrically with secondary generalization. In contrast, DYT-THAP1 may initiate in the upper part of the body, involving cranio–cervical districts, speech difficulties, and the upper limbs, with successive generalizations [73]. If DYT-TOR1A begins in the upper limbs, it tends to be focal. Focal and segmental isolated dystonia are more likely caused by DYT-GNAL and DYT-ANO3. These two forms of dystonia typically begin at the cervical level and may cause head tremor [70]. DYT-GNAL may be suspected if age at onset is in early-late adulthood. In case of early involvement of craniofacial muscles with laryngeal dystonia and speech difficulties, with secondary generalization involving the arms at younger ages, DYT-ANO3 becomes more probable [70]. Another peculiar form of isolated dystonia with focal distribution involving the cervical district and causing spasmodic dysphonia is caused by DYT-TUBB4A. This focal form may successively evolve into a generalized dystonia [74]. Regarding the temporal pattern, except for the last-mentioned dystonia, all the other isolated dystonia follows a persistent temporal pattern. Associated features may guide the clinician in the differential diagnosis. The presence of additional phenotypic characteristic, such as microcephaly, short stature, intellectual disability, abnormal eye movements, myoclonus, dysmorphisms, and psychiatric symptoms, may be suggestive of DYT-KMT2B [70]. Thin face, body habitus, and hobby horse gait are described in the DYT-TUBB4A [75]. None of the isolated forms of dystonia respond to L-Dopa; DYT-TOR1A, DYT-THAP1, DYT-ANO3, DYT-KMT2B, and DYT-HPCA may respond to anticholinergics [70]. Response to alcohol is described in DYT-GNAL and DYT-TUBB4A. It is important to define the genetic etiology of the dystonia because response to DBS varies according to the genetic conditions, and this is an important prognostic factor to be considered when selecting patients for advanced therapy. Indeed, is well known that DYT-TOR1A, DYT-THAP1, DYT-ANO3, DYT-GNAL, and DYT-KMT2B show a good response to DBS with a target in the GPi, unlike the other forms of isolated dystonia [76,77,78,79]. Brain imaging is not conclusive in distinguishing between the several forms of isolated dystonia, as the sole characteristic described is pallidal hypointensity in DYT-KMT2B [70]. 

Combined dystonia is characterized by the coexistence of another movement disorder in addition to dystonia. The association of dystonia with parkinsonism defines dystonia–parkinsonism. The monogenic forms of dystonia–parkinsonism are DYT/PARK-GCH1, DYT/PARK-TH, DYT/PARK-TAF1, and DYT/PARK-ATP1A3 [71]. Contrary to what has been observed for isolated dystonia, combined dystonia recognizes a different mode of inheritance: autosomal dominant inheritance is characteristic of DYT/PARK-GCH1 and DYT/PARK-ATP1A3, while autosomal recessive inheritance is typical of DYT/PARK-TH. X-linked transmission characterizes DYT/PARK-TAF1 (also known as Lubag syndrome). Among this, it is of paramount importance to diagnose the dopa-responsive dystonia, DYT/PARK-GCH1. Indeed, patients have excellent and sustained response to L-Dopa [80]. Another form of combined dystonia with response to L-Dopa is DYT/PARK-TH. These two forms of dystonia–parkinsonism may be differentiated according to age of onset, as DYT/PARK-GCH1 begins in infancy/childhood, while DYT/PARK-TH may initiate in infancy. Moreover, diurnal fluctuations of parkinsonian symptoms due to circadian variations in dopamine concentration are more pronounced in DYT/PARK-GCH1 than in DYT/PARK-TH [80]. An adjunctive feature may help in differential diagnosis among the two forms: the presence of hypotonia is suggestive of DYT/PARK-TH, while in DYT/PARK-GCH1 hyperreflexia has been described [81]. The coexistence of non-motor features orients towards the diagnosis of DYT/PARK-GCH1, while a more complex clinical picture, with autonomic disturbances, ptosis, and oculogyric crisis is suggestive of DYT/PARK-TH. In both forms, dystonia begins as focal with subsequent generalization [82,83,84,85]. 

DYT/PARK TAF1 differs from the previous mentioned strains for the age of onset, body distribution of dystonia, and neuroimaging. This form of combined dystonia begins in early to late adulthood and, contrary to DYT/PARK-GCH1 that begins with foot dystonia and then progress cranially, DYT/PARK TAF1 involves mainly the upper body, with characteristic jaw opening dystonia and bulbar involvement. Another difference with respect to the dopa-responsive dystonia is the absence of diurnal fluctuation. Brain imaging shows striatal atrophy and pallidum volume loss, considered an expression of the neurodegenerative nature of the disease. This form recognizes an X-linked transmission, hence is more frequent in males [86,87,88,89]. Abrupt onset, fluctuating course, psychiatric features, and postural instability may raise suspicion of DYT/PARK-ATP1A3. This disease begins with dystonic spasms, usually following a provoking event (fever, infection, childbirth, alcohol binging, fall, excessive exercise, heat exposure, and psychological stress), with a plateau within 30–60 days of disease onset, with no significant improvement [90]. Dystonia begins in limbs and develops with a characteristic rostrocaudal gradient, cranial symptoms being more severe than upper limbs and lower limbs [91].

Combined dystonia also encompasses dystonia associated with myoclonus and other hyperkinetic disorders. To date, two forms of dystonia–myoclonus have received confirmations: DYT-SGCE and DYT-KCTD17. These diseases have several features in common: age of onset is in the first or second decade of life, myoclonic jerks involve the upper body, and in DYT-SGCE also the neck may be involved. In both diseases, dystonia affects the upper part of the body, with involvement of upper limbs and the cranio-cervical region. If in DYT-SGCE myoclonic jerks dominates the clinical picture, in DYT-KCTD17 dystonia seems to be the prominent feature. Interestingly, DYT-SGCE myoclonic symptoms respond to alcohol, while in DYT-KCTD17 this response is absent [71,92]. 

Dystonia may coexist with other hyperkinetic disorders, such as chorea, as observed in several forms of complex dystonia. Marras et al. [69] also classify CHOR/DYT-ADCY5 as combined dystonia. This disease is characterized by a plethora of hyperkinetic disorders, such as chorea, dystonia, and myoclonus, beginning in early childhood and with a characteristic fluctuating or paroxysmal course. Interestingly, symptoms do not disappear during sleep, resulting in significant disturbances, and may respond to caffeine [93,94]. Response to DBS is lower than in other form of monogenic dystonia [76].

### Genetic Testing and Genetic Counseling

According to the EFNS dystonia guidelines, genetic testing is not sufficient to make a diagnosis of dystonia in the absence of clinical features suggestive of dystonia [95]. Therefore, the clinical picture should orient the decision to carry out genetic testing [96,97,98].

The previously mentioned guidelines recommend, with a level B of evidence, the DYT-TOR1A testing for patients with limb-onset, primary dystonia with onset before age 30 [98], and in those with onset after age 30 if they have an affected relative with early-onset dystonia [98]. Guidelines do not recommend DYT-TOR1A testing in asymptomatic individuals in dystonia families as a good practice point. After exclusion of DYT-TOR1A, in early-onset dystonia or familial dystonia with cranio-cervical predominance, DYT-THAP1 testing is recommended [73]. It is considered a good practice point to conduct a diagnostic levodopa trial in every patient with early-onset dystonia without an alternative diagnosis [99]. Individuals with early-onset myoclonus affecting the arms or neck, particularly if positive for autosomal-dominant inheritance and if triggered by action, should be tested for the DYT-SGCE gene [100].

In clinical practice, genetic testing consists of of using predefined panels for dystonia. The whole-exome sequencing (WES) is also a resource to consider; however, it is expensive and requires a long time. Zech et al. [101] proposed an algorithm to predict diagnostic success rate of WES in individuals with dystonia. This algorithm assigns a score to three clinical characteristics:

- Age at onset (0–20 years: score 2; >21 years: score 0),
- Body distribution (generalized or segmental: score 1; focal: score 0),
- Dystonia category (complex dystonia: score 2; combined dystonia: score 1; isolated dystonia: score 0).

Summary scores range from 0 to 5 and predict the diagnostic success rate of WES in individuals with dystonia. If the score is three, the sensitivity is 96% and the specificity is 62%; if the score is five, the sensitivity is 62% and the specificity is 86%. Hence, if the score is equal to or higher than three, whole-exome sequencing is recommended [101].

An extensive discussion about genetic counseling goes beyond the scope of this review. The main concept to underscore is that genetic counseling depends largely on the determination of the mode of inheritance of a specific cause of an inherited dystonia in an individual (i.e., autosomal dominant, autosomal recessive, mitochondrial, X-linked inheritance). According to the inheritance, Table 3 describes all the possible diseases [4].

Moreover, penetrance must be considered because of the influence of the phenotypic expression of dystonia [102]. For example, for two hereditary forms of dystonia, mechanisms affecting penetrance have been identified:

- DYT-SGCE dystonia has maternal imprinting of the gene, meaning that the dystonia-myoclonus only manifests when SGCE pathogenic variants are paternally inherited [103].
- DYT-TOR1A has a reduced penetrance of the GAG deletion in TOR1A, from about 35% to 3% in individuals who also have a heterozygous NM_000113.2:646G>C (p.Asp216His) variant in TOR1A on the other allele [72].

Genetic counseling should be offered to the patients and the family by qualified personnel and, according to the EFNS dystonia guideline, is recommended [95].

## 4. Discussion

**Figure 3:**
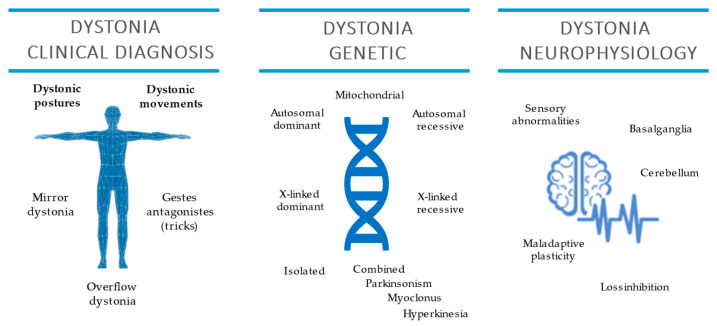
Dystonia clinical diagnosis and genetic and clinical neurophysiology features.

The present review summarized the possible contribution of clinical neurophysiology and genetic testing to clinical examination for dystonia diagnosis (Figure 3).

However, dystonia diagnosis is still based on clinical examination conducted by physicians with expertise in movement disorders. The clinical diagnosis should be based on the observations of two core characteristics and of adjunctive features [1]. According to the EFNS dystonia guidelines, a neurophysiological test may help diagnosis despite low evidence (class IV), hence further and proper studies are needed [95]. However, the role of neurophysiology is not marginal, being an important resource to enlighten the pathophysiology of dystonia (Table 1). Among neurophysiological alterations observed in dystonia, sensitivity, specificity, and positive predictive value have been evaluated only for STDT. That is pathologically increased in patients affected by cervical dystonia compared to patients affected by essential tremor [37]. Neurophysiology also represents an excellent support for the therapy of dystonia in the case of EMG-guided botulinum toxin injection. In future applications, neurophysiology could guide adaptive DBS. Indeed, LFP recorded in GPi could be used as input signals to modulate stimulation parameters as currently used for Parkinson’s disease [104].

Once dystonia has been clinically diagnosed, the definition of the etiology is needed [1]. The etiological diagnosis of dystonia cannot ignore the role of genetics testing. Several genes have been described as causes of isolated, combined, or complex forms of dystonia (see Table 2). Regarding isolated dystonia, age at onset, body distribution (focal, segmental or generalized), and associated features may orient the clinicians towards a specific form of monogenic dystonia. In combined dystonia, the second most represented movement disorder, the clinical picture guides the clinician in the direction of dystonia associated with parkinsonism, myoclonus, or other hyperkinesia. The choice to request WES to reach a diagnosis should be carefully considered when panels for dystonia fail to detect causative mutations. Zech et al. proposed an interesting and feasible algorithm to predict diagnostic success rate of WES, according to dystonia characteristics [101]. The algorithm considers tree items (age at onset, body distribution, dystonia category) and assigns a score to each one. If the summary score is equal or higher than three, WES is recommended because of a high probability to identify causative mutations.

Considering the inheritance mode and the risk of transmission of the disease in the context of the same family, genetic counseling should be offered to the patients and a multidisciplinary approach involving geneticists, psychologist is desirable.

## Figures and Tables

**Table 1:** Main neurophysiological findings in dystonia.

	Neurophysiological Test	Results	Accuracy	Ref.
**Loss inhibition**	EMG	Prolonged bursts Co-contraction agonist and antagonist muscles Overflow to other muscles	NA	[23]
	Spinal cord reciprocal inhibition	Reduced reciprocal inhibition	NA	[24]
	Blink reflex recovery cycle	Reduced inhibition of R2 component	NA	[25]
	Short latency trigemino-sternocleidomastoid response	Impairment of the trigemino-cervical reflex	NA	[26]
	SICI	Reduced in most studies	NA	[27,28,29]
	IHI	Loss of suppression	NA	[30]
	SP	Reduced	NA	[31,32]
**Cerebelum**	EBCC	Impaired in primary focal dystonia Normal in DYT-TOR1A and DYT-THAP1 dystonia	NA	[33,34]
	CBI	Absent	NA	[35]
**Sensory Abnormalities**	GOT	Increased SD threshold in blepharospasm, CD, FHD Normal in DYT-TOR1A	NA	[36]
	STDT	Abnormally increased STDT (higher in CD patients with tremor). No statistical differences between CD and PD	CD compared to ET: ≤67 ms: 100% Sens 100% NPV ≥120 ms 100% Spec, 100% PPV	[37]
	TVR	Abnormally increased	NA	[38]
**Maladaptive Plasticity**	PAS	Abnormally increased in dystonic patients Normal in functional dystonia and DYT-TOR1A carrier	NA	[39]
	HF-RSS	Reduced inhibition	NA	[40]
**Basal Ganglia**	LFP recordings (GPi)	Synchronized activities in 4–10 Hz band	NA	[41,42]

Legend: CBI: cerebellar brain inhibition; CD: cervical dystonia; EBCC: eyeblink classic conditioning; EMG: electromyography; ET: essential tremor; GOT: grating orientation task; HF-RSS: high-frequency repetitive somatosensory stimulation; IHI: inter-hemispheric inhibition; NA: not available. NPV: negative predictive value; PAS: paired associative stimulation; PD: Parkinson’s disease; PPV: positive predictive value; Sens: sensitivity; SD: spatial discrimination; SICI: short intra-cortical inhibition; SP: silent period; Spec: specificity; STDT: somatosensory temporal discrimination threshold; TVR: tonic vibration reflex.

**Table 2:** Isolated and combined genetic types of dystonia.

Phenotype		Gene/ Locus	Inheritance/Penetrance	OMIM	Age of Onset	Body Distribution	Temporal Pattern	Associated Features	Drugs Response			DBS Response	Brain Imaging Findings	References
									Dopa	Other Drugs	Alcohol			
Isolated		TOR1A/ DYT 1	AD/ Reduced	128100	Childhood-Adolescence-Early adulthood	Generalized	Persistent	none	No	Anticholinergics	No	Good	None	[70]
		THAP1/ DYT 6	AD/48%	602629	Childhood-Adolescence	Segmental-generalized	Persistent	Laryngeal dystonia/dysarthria/dysphonia	No	Anricholinergics	No	Variable	None	[70,73]
		ANO3/ DYT 24	AD/NA	615034	Infancy/childhood, early and late adulthood	Focal-Segmental	Persistent	Tremor	Yes	Anticholinergics/Antiepileptics	No	Good	None	[70,78]
		GNAL/ DYT 25	AD/High	615073	Early adulthood-Late adulthood	Focal-segmental-occasionaly generalized	Persistent	none	No	No	Yes	Good	None	[70]
		KMT2B/ DYT 28	AD/ Incomplete	617284	Infancy-Childhood-Adolescence	Generalized	Persistent	Nonmotor signs, neurodevelopemental disorders, Dysmorphisms, Psychiatric symptoms,	No	Anticholinergics	No	Good	Pallidal hypointensity	[70]
		HPCA/ DYT2	AR	224500	Infancy/childhood	Generalized	Persistent	Psychiatric features, cognitive impairment, dystonic tremor	No	Anticholinergics	No	Not know	None	[70]
		TUBB4A/ DYT 4	AD/High	128101	Childhood-Adolescence	Focal-generalized	Spasmodic dysphonia	Thin face-body habitus-hobby horse gait	No	No	Yes	Not known	None	[74,75]
		PRKRA/ DYT 16	AR/NA	612067	Infancy-Childhood-Adolescence	Generalized	Persistent	Parkinsonism, Hyperreflexia	No	No	No	Not known	None	[70]
CombinedKC	Parkinsonism	GCH1/ DYT 5a	AD/50%	128230	Infancy-Childhood	Mostly generalized	Diurnal fluctuations	Parkinsonism-spasticity-non motor features	Yes	None	No	Not known	None	[80,81]
		TH/ DYT 5b	AR/NA	605407	Infancy	Mostly generalized	Diurnal fluctuations	Parkinsonism-ptosis- hypotonia-autonomic disturbances, oculogyric crises, developmental delay	Yes	None	No	Not known	None	[82,83,84,85]
		TAF1/ DYT 3	XL/Full	314250	Early adulthood-Late adulthood	Generalized	Persistent	Parkinsonism, jaw opening dystonia, bulbar involvement, striatal toe	No	None	No	Variable	Stiatal atrophy and pallidum volume loss in pallidum	[86,87,88,89]
		ATP1A3/ DYT 12	AD/ Incomplete	128235	Adolescence-Early adulthood	Generalized-Segmental	Persistent	Abrupt onset, Fluctuating course, Parkinsonism, Postural instability, Psychiatric features	No	None	No	Not known	None	[85,90,91]
	Myoclonus	SGCE DYT 11	AD/ Reduced (maternal imprinting)	159900	Childhood-Adolescence	Focal-segmental	Persistent	Myoclonic jerks mainly of the neck, prominent psychiatric features	No	None	Yes	Variable	None	[71]
		KCTD17 DYT26	AD/NA	616398	Childhood-Adolescence	Focal-segmental	Persistent	Myoclonus of upper limbs, psychiatric features,	No	None	No	Good	None	[92]
	Hyperkinesia	ADCY5	AD/NA	600293	Childhood	Focal-segmental-generalized	Paroxysmal worsening	Generalized choreoathetosis, Facial dyskinesia, myoclonus, learning difficulties, behavioral abnormalities	No	Caffeine	No	Variable	None	[76,93,94]

Legend: AD autosomic dominant, AR autosomic recessive, XL X Linked, NA not available.

**Table 3:** Inherited causes of dystonia.

Autosomal Dominant	
**Disease**	**OMIM Code**
- Oppenheim dystonia (DYT-TOR1A)	#128100
- Childhood and adult onset-familial cranial limb dystonia (DYT-THAP1)	#602629
- Dopa-responsive dystonia (DYT/PARK-GCH1)	#128230
- Rapid-onset dystonia–parkinsonism (DYT/PARK-ATP1A3)	#128235
- Myoclonus–dystonia (DYT-SGCE)	#159900
- Neuroferritinopathy (NBIA/CHOREA-FTL)	#606159
- Dentatorubral-pallidoluysian atrophy	#125370
- Huntington’s disease	#143100
- Machado–Joseph disease (SCA-ATXN3)	#109150
- Creutzfeldt–Jakob disease	#123400
- Primary Familial Brain Calcification	#213600
- Myclonic-dystonia 26 (DYT-26)	#616398
- Dystonia-28 (DYT-KMT2B)	#617284
- Dystonia-30 (DYT-30)	#619291
- Dystonia-33 (DYT-33)	#619687
- Dystonia-25 (DYT-GNAL)	#615073
- Dystonia-24 (DYT-ANO3)	#615034
- Dystonia-4 (DYT-TUBB4A)	#129101
- Dystonia-26 (DYT-KCTD17)	#616398
- Dyskinesia with orofacial involvement (CHOR/DYT-ADCY5)	#606703
Autosomal recessive:	
- Wilson disease	#277900
- Neurodegeneration with brain iron accumulation type 1 (NBIA/DYT-PANK2)	#234200
- Neurodegeneration with brain iron accumulation type 2, infantile neuroaxonal dystrophy (NBIA/DYT/PARK-PLA2G6)	#610217
- Aceruloplasminemia (NBIA/DYT/PARK-C)	#604290
- Fatty acid hydroxylase-associated neurodegeneration (FAHN) (HSP/NBIA-FA2H)	#612319
- Early-onset parkinsonism (PARK-Parkin) (PARK-PINK1)	#608309
- Aromatic-L-amino acid decarboxylase (DYT-DDC)	#608643
- Early-onset dystonia with parkinsonism (DYT-PRKRA)	#612067
- Niemann–Pick type C	#257220
- Juvenile neuronal ceroid-lipofuscinosis (Batten disease)	#204200
- GM1 gangliosidosis (DYT/PARK-GLB1) type III, chronic/adult form	#230500
- GM2 gangliosidosis	#272750
- Metachromatic leukodystrophy	#250100
- Homocystinuria	#277400
- Glutaric acidemia (DYT/CHOR-GCDH)	#231670
- Methylmalonic aciduria (DYT/CHOR-MUT)	#251000
- Hartnup disease	#234500
- Ataxia telangiectasia	#208900
- Friedreich ataxia	#229300
- Neuroacanthocytosis	#200150
- Dopa-responsive dystonia (DYT/PARK-TH)	#605407
- Neuronal intranuclear hyaline inclusion disease	#603472
- Hereditary spastic paraplegia (HSP-SPG7)	#607259
- Sjögren–Larsson syndrome (ichthyosis, spasticity, intellectual disability)	#270200
- Biotin-responsive basal ganglia disease (DYT-SLC19A3)	#607483
- Dystonia musculorum deformans 2 (DYT-HPCA)	#224500
- Zech-boesch syndrom (DYT-31)	#619565
X-linked recessive:	
- Dystonia-parkinsonism or Lubag syndrome (DYT/PARK-TAF1)	#314250
- Lesch-Nyhan syndrome (DYT/CHOR-HPRT)	#300322
- Mohr-Tranebjaerg syndrome (Deafness–dystonia syndrome) (DYT-TIMM8A)	#304700
X-linked dominant	
- Rett syndrome	#312750
Mitochondrial	
- Leigh syndrome	#256000
- Leber’s hereditary ocular neuropathy plus dystonia (DYT-mt-ND6)	#500001

Legend: OMIM code = Online Mendelian Inheritance in Man code (reproduced under the terms and conditions of the Creative Commons Attribution (CC BY) license from [4]).
